# Immediate postoperative effects of cytoreductive surgery with hyperthermic intraperitoneal chemotherapy using carboplatin on peritoneal tissue inflammatory and ischemic responses: an explorative porcine study

**DOI:** 10.1515/pp-2025-0001

**Published:** 2025-04-22

**Authors:** Elisabeth Krogsgaard Petersen, Mats Bue, Christina Harlev, Andrea René Jørgensen, Pelle Hanberg, Lone Kjeld Petersen, Maiken Stilling

**Affiliations:** Department of Clinical Medicine, 1006 Aarhus University, Aarhus, Denmark; Aarhus Denmark Microdialysis Research (ADMIRE), Orthopedic Research Laboratory, Aarhus University Hospital, Aarhus, Denmark; Department of Orthopedic Surgery, Aarhus University Hospital, Aarhus, Denmark; Department of Gynecology and Obstetrics, Odense University Hospital, Odense, Denmark; Department of Clinical Medicine, University of Southern Denmark, Odense, Denmark

**Keywords:** cytoreductive surgery, hyperthermic intraperitoneal chemotherapy (HIPEC), carboplatin, lactate, ischemic metabolites, acute inflammatory protein markers

## Abstract

**Objectives:**

This explorative porcine study investigated the immediate postoperative response of inflammatory protein markers and ischemic metabolites in peritoneal abdominal wall tissue during and after hyperthermic intraperitoneal chemotherapy (HIPEC) assessed by microdialysis.

**Methods:**

Eight cancer-free female pigs underwent imitation cytoreductive surgery (CRS) followed by HIPEC (90 min) using a carboplatin dosage of 800 mg/m^2^. A subperitoneal microdialysis catheter was placed in the abdominal wall to sample inflammatory protein markers and ischemic metabolites. During and after HIPEC, dialysates and blood samples were collected over 8 h. Inflammatory protein expression levels were quantified using Proximity Extension Assay (Olink Target 96 Inflammation panel). In addition, concentrations of ischemic metabolites were quantified using a CMA600 microdialysis analyzer.

**Results:**

An immediate response of 27 proteins, including proteins from the TNF family, early inflammatory chemokines, pro- and anti-inflammatory proteins, was found within the first 30 min after HIPEC initiation. This was followed by a modest and relatively constant inflammatory response. Glycerol concentrations tended to decrease during the sampling period, while glucose showed more stable levels. Constant hyperlactatemia exceeding the plasma levels was seen during and after HIPEC, with no significant tissue ischemia as indicated by the lactate/pyruvate ratios.

**Conclusions:**

In a porcine model, CRS with HIPEC leads to an immediate and relatively constant increased inflammatory and ischemic peritoneal tissue short-time response. The explorative findings contribute to the debate on the value of HIPEC in combination with CRS and call for future studies to further investigate the inflammatory and metabolic tissue responses.

## Introduction

In recent years, the focus on combining cytoreductive surgery (CRS) with hyperthermic intraperitoneal chemotherapy (HIPEC) in treating peritoneal malignancies has increased [1], 2]. CRS is defined as removing all visible tumor tissue within the abdomen, while HIPEC intends to eradicate the remaining microscopic disease by circulating a heated high-concentration chemotherapeutic solution within the abdominal cavity [3]. Thus, the patients undergo a complicated and prolonged procedure, often followed by elective admission to the intensive care unit with a morbidity rate of 12–60 % and a mortality rate of 0.9–5.8 % in the immediate postoperative period [4]. The most prevalent postoperative complications are gastrointestinal, with paralytic ileus being the most common [4]. The etiology of the postoperative complications of CRS with HIPEC is poorly understood.

Systemic and peritoneal fluid inflammatory cytokine responses have been studied 1–3 days after CRS with HIPEC in colorectal cancer patients with peritoneal metastases, showing a significant inflammatory response in the peritoneal fluid compared to plasma [5]. In general, tissue inflammatory responses are induced by exogenous and/or endogenous factors. Endogenous factors are signals produced locally due to stressed, damaged, or malfunctioning tissue [6]. Hyperthermia and high local concentrations of chemotherapy combined with extended surgery procedures can lead to a comprehensive immediate exogenous response [7], 8]. Innate inflammatory mechanisms are believed to play a significant role in developing postoperative complications [9].

Compared to other major abdominal cancer surgeries, significantly increased systemic plasma lactate levels have been shown in patients undergoing CRS with HIPEC [10], 11]. Increased systemic lactate levels have been investigated as an early marker of postoperative complications and morbidity following CRS with HIPEC [10], 12]. However, the extent of the local intra-abdominal tissue response and the correlation between the local and systemic response is unknown [10].

In an exploratory porcine setup, we performed CRS-imitated surgery. Subsequently, we applied microdialysis to dynamically assess inflammatory protein markers and ischemic metabolites in peritoneal abdominal wall tissue during and after HIPEC, aiming to evaluate the local tissues’ immediate postoperative response. Understanding the immediate effects of surgical procedures at the molecular and cellular levels is critical for elucidating the development of postoperative complications associated with CRS, HIPEC, and their combined application. Moreover, this knowledge may inform potential optimizations of these procedures.

## Materials and methods

The study was conducted at the Institute of Clinical Medicine, Aarhus University Hospital, Denmark. It was approved by the Danish Animal Experiments Inspectorate and carried out according to existing laws and institutional policies (license No. 2017/15-0201-01184) and to the ARRIVE guidelines. Attending to the three Rs of research with animals, a study on carboplatin pharmacokinetics conducted with the same animals was previously published [13].

### Study procedures

#### Microdialysis

Microdialysis is based on diffusion over a semipermeable membrane [14]. This allows for continuous and simultaneous sampling of small sample solutions in vials mirroring the extracellular fluid in target tissues. Due to a constant perfusion of the catheter, total equilibrium will never occur. The sample will only represent a fraction of the absolute tissue concentration called the relative recovery [15].

Microdialysis equipment (M Dialysis AB, Stockholm, Sweden) comprised type 71 microdialysis catheters (membrane length: 30 mm; molecular cut-off: 100 kilo-Dalton), SI-2 splitable introducers, and microdialysis 107 pumps, at a flow rate of 1 μL/min using CNS dextran as the perfusion fluid, per manufacturer recommendations.

#### Animals, anesthesia, surgical, and HIPEC procedures

Eight cancer-free female pigs (Danish Landrace Breed, weight 71–83 kg, age 5 months) were included. During the experiment, the pigs were kept under general anesthesia with a continuous infusion of propofol (400–600 mg/h, Fresenius Kabi, Bad Homburg, Germany) and fentanyl (1.2–1.5 mg/h, B. Braun, Melsungen, Germany). Arterial pH was kept within 7.3–7.5. Rectal temperature was kept within 36.5–39.0 °C. All pigs were euthanized at the end of the experiment by an overdose of intravenously administered pentobarbital. All animals underwent an imitation of CRS by bilateral salpingo-oophorectomy, hysterectomy, total omentectomy, and peritoneal stripping with the removal of 10×10 cm peritoneal tissue from the left anterior abdominal wall to imitate the removal of peritoneal metastasis.

After the surgical procedure, a microdialysis catheter was placed subperitoneally in the right abdominal wall. The location was chosen, as in ovarian cancer, the peritoneum is often the site of metastatic involvement, and general tissue effects from surgical and HIPEC procedures throughout the abdomen were expected. Using an SI-2 splitable introducer inserted underneath the peritoneal layer, the microdialysis catheter was placed into the introducer lumen directly following the removal of the needle inside the introducer. Next, the introducer was carefully split and removed, leaving only the catheter membrane sufficiently within the tissue and at a distance to the tissue entry of at least 2 cm. The catheter was secured to the tissue with a single suture. After placement, the microdialysis catheter was perfused with CNS dextran, and 30 min of tissue equilibration was allowed.

The HIPEC procedure was performed with a single dose of carboplatin 800 mg/m^2^ (10 mg/mL, Fresenius Kabi, Bad Homburg, Germany) circulating in the abdominal cavity and kept at a temperature of 40–42 °C for 90 min. Details regarding CRS and HIPEC procedures can be found elsewhere [13].

#### Sampling procedures

**Figure 1:**
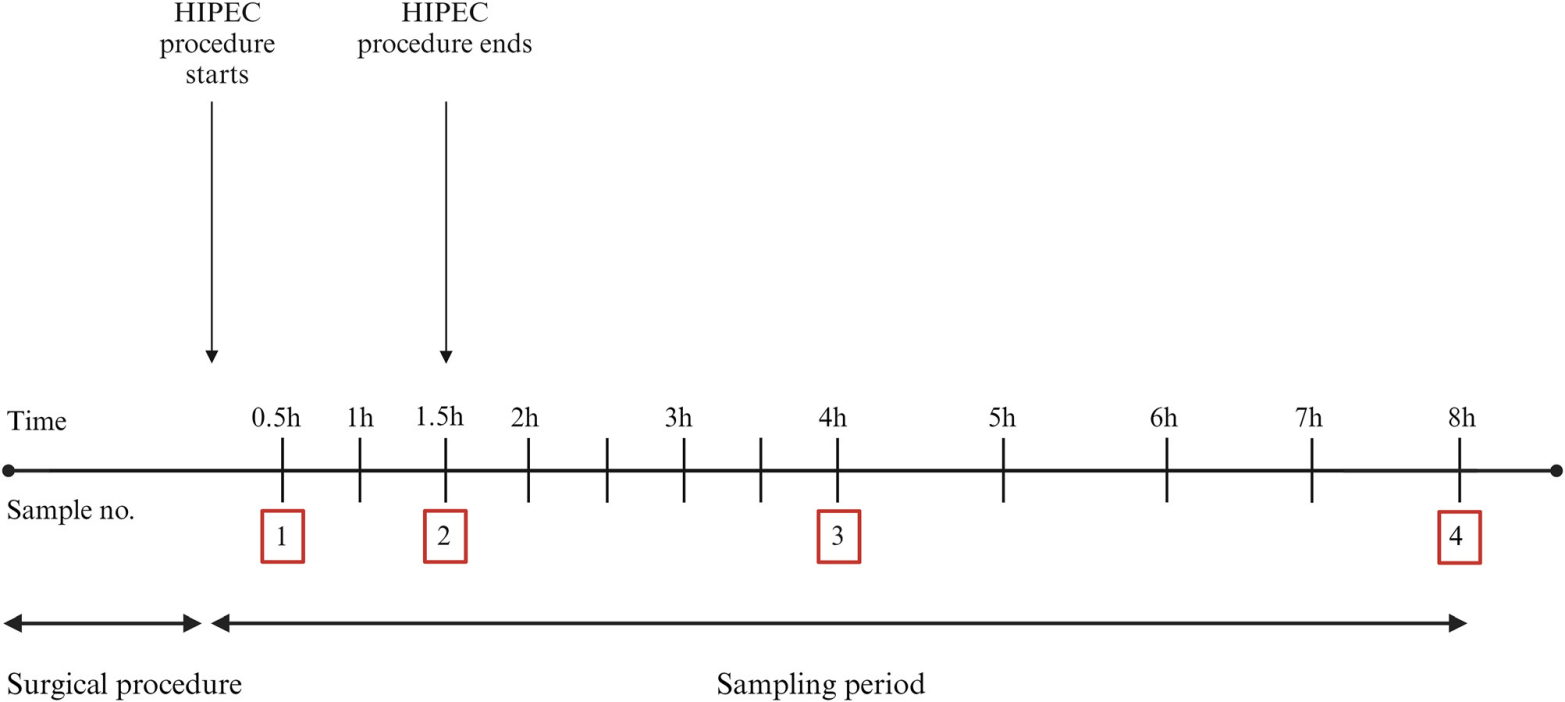
Timeline illustrating the sampling process. The time and sample number are given above and below the timetable, respectively. Created with BioRender.

Time zero of the 8-hour sampling period was marked by HIPEC initiation. The following dialysate samples were taken: Sample no. 1 (time interval: 0–30 min), 2 (time interval: 60–90 min), 3 (time interval: 210–240 min), and 4 (time interval: 420–480 min) (Figure 1). Arterial blood gas samples were taken once every hour.

### Inflammatory protein markers

All analyses of the inflammatory data were done by BioXpedia A/S (Aarhus, Denmark). Olink Target 96 Inflammation panel (version 3023) from Olink Proteomics (Uppsala, Sweden) was used to evaluate inflammation protein markers. The panel used Proximity Extension Assay (PEA), enabling multiplex immunoassay polymerase chain reaction (PCR) to measure 92 inflammation-associated proteins across 88 samples simultaneously. From each sample, 1 µL was used for each assay. Each PEA measuring a specific protein contains two antibodies, each labeled with one oligo that is complementary to each other. When both antibodies bind to the target protein simultaneously, the oligos are in proximity, enabling hybridization and extension, forming a double-stranded oligonucleotide. A DNA polymerase is added to generate PCR templates for oligonucleotide amplification, which is quantified using microfluidic real-time quantitative PCR (96.96 Dynamic Array™ Integrated Fluidic Circuit (IFC) Fluidigm BioMark, South San Francisco, California, USA). Each assay also contains two internal controls to monitor all steps of the PEA technology procedure. In addition, three replicates of Olink negative controls are used to measure background protein levels and assay-specific limit of detection (LOD), and three replicates of Olink three interplate controls to calculate normalized protein expression (NPX).

Before statistical analysis, quality control was performed using the standard Olink QC software and in-house quality control. If the missing data frequency of a sample or protein was lower than 10 %, the missing data points were imputed using the k-nearest neighbors method for each cohort (the groups of each sample time point) [16]. If the missing data frequency of a sample or protein was higher than 10 %, this sample or protein was excluded from further statistical analysis. As a result, no proteins or samples had to be excluded, but data were imputed for the proteins CX3CL1 and Flt3L.

Data are given as arbitrary log2-transformed units referred to as NPX; thus, higher NPX values correlate to higher protein expression levels. Public bioinformatic databases, UniProt, STRING (version 12.0), and Gene Ontology (GO) (biological processes) were reviewed in the biological interpretation [17], [18], [19], [20].

Since baseline samples before CRS and HIPEC initiation were not obtained, the protein measurement frequency was determined in the first samples collected from 0 to 30 min to indicate the immediate inflammatory response. Above 70 % measurement frequency was used as an explorative cutoff for the proteins suggesting the immediate response.

### Ischemic metabolites

The consequence of hypoxia is cellular anaerobic glycolysis, causing decreased plasma glucose levels and increased glycerol levels due to cell damage. During anaerobic processes, pyruvate is converted to lactate, decreasing pyruvate and increasing lactate levels and the lactate/pyruvate (L/P) ratio. Hyperlactatemia in plasma is defined as lactate levels between 3 and 4 mmol/L, while an L/P ratio above 25 indicates significant tissue ischemia [21].

Glucose, glycerol, lactate, and pyruvate levels were quantified using a CMA600 microdialysis analyzer using Reagent Set A from M Dialysis AB (Stockholm, Sweden). Systemic lactate levels were measured from arterial blood gas samples (ABL 90 FLEX PLUS, Radiometer Medical ApS, Brønshøj, Denmark).

### Statistics

All measured protein expression levels and ischemic metabolite concentrations were applied to the midpoint of the sampling intervals.

To evaluate the differences in protein expression levels, comparisons between groups of the first time point and the remaining three time points were performed. Differential expression between time points was tested using *t*-tests for independent samples. If the central limit theorem did not apply because the group had less than 30 samples, normal distribution was checked using the Shapiro–Wilk test. If a group was not normally distributed, a Wilcoxon rank sum test or a signed rank test was conducted [22]. Furthermore, the fold change was calculated on a linear scale as the geometric mean of the first group divided by the geometric mean of another group. The p-Values were corrected for multiple testing using the Benjamini–Hochberg method [23]. All statistical analyses in this study were made in R (version 4.1.3). A p-value<0.05 was considered statistically significant.

The ischemic metabolites data were analyzed on a logarithmic scale using STATA (v. 18, StataCorp LLC, College Station, TX, USA). The assumption of normal distribution was assessed using Quantile–Quantile (QQ) plots and Bland–Altman plots. The ischemic concentration differences between the time points were compared using a paired *t*-test.

Given the exploratory nature of this study and the ethical considerations inherent in using an animal model, our sample size was carefully chosen to balance scientific validity with ethical responsibility. To our knowledge, this is the first study of its type providing pilot insights, wherefore choosing a small sample size is generally advised in animal studies [24].

## Results

All pigs completed the study. Malfunction was experienced for one catheter. Displacement of another catheter outside the peritoneum was observed after HIPEC; therefore, sample no. 3 and 4 were omitted from this catheter. In total, 26 samples were collected.

### Inflammation protein markers

Approximately 90 % of measurements were below LOD, so 19 proteins were excluded. FIt3L did not pass the quality control, leaving 72 proteins for further analysis. Supplementary Table 1 shows an overview of all proteins.

#### The explorative immediate inflammatory response

**Figure 2:**
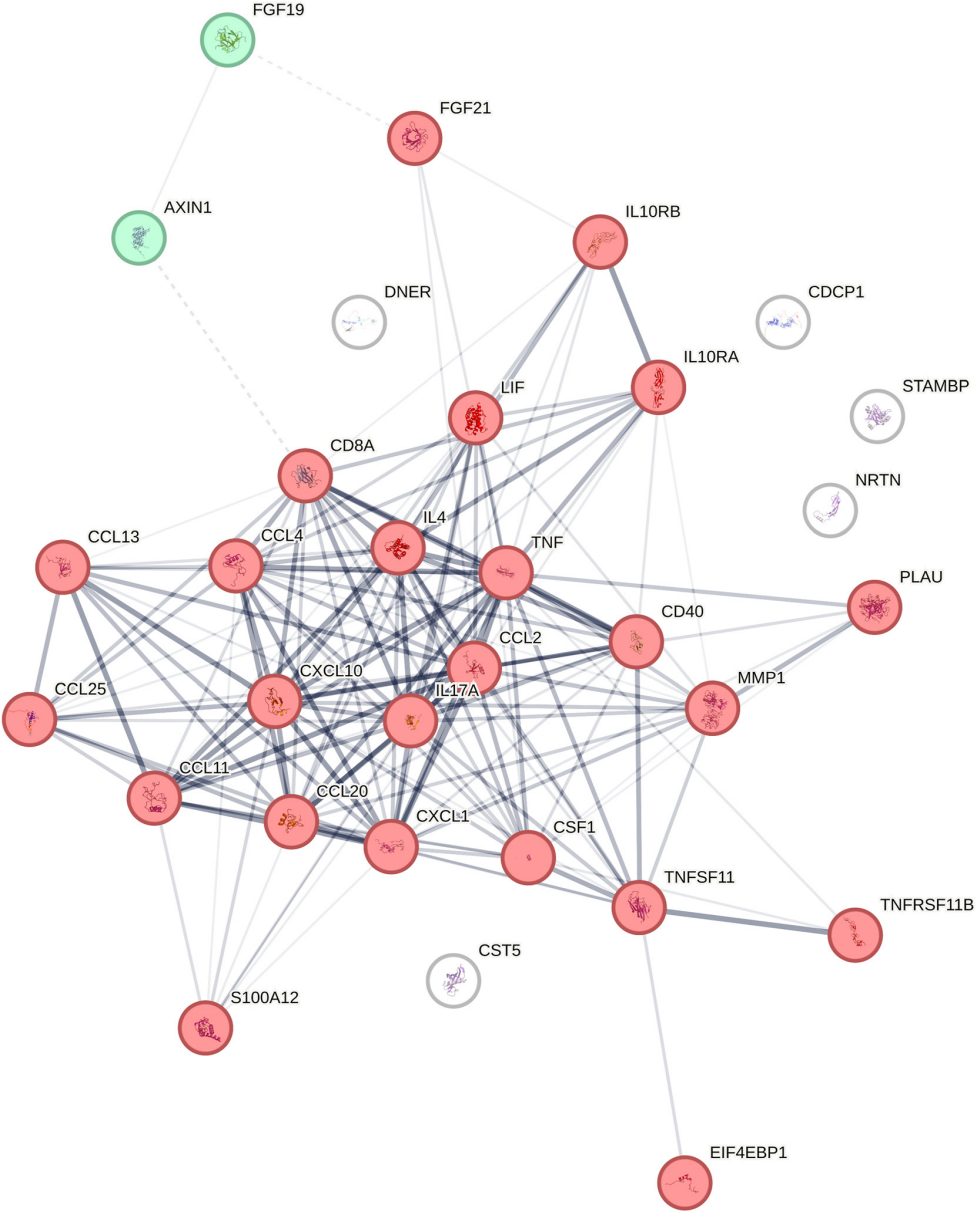
PPI STRING network including 34 of the 35 proteins of interest. Edges mark protein–protein associations by confidence, meaning the thickness of the lines indicates the degree of confidence of the predicted association. Two clusters are present: a red cluster of 24 nodes, and a green cluster of 2 nodes. Created with STRING 12.0.

A total of 35 of the 72 analyzed proteins reached >70 % measured frequency. A protein–protein interaction (PPI) STRING network was conducted to explore any biological associations between the proteins. Figure 2 presents the PPI STRING network with medium confidence (0.400), meaning that >40 % of the visualized interactions in the PPI STRING network are significant. The proteins were clustered in the STRING network by the recommended Markov Cluster Algorithm-clustering (generally referred to as MCL clustering) with an inflation parameter of 1.7 [25]. A large cluster of 24 nodes describes viral protein interaction with cytokines and cytokine receptors, myeloid leukocyte migration, and IL-10 signaling. Some proteins, e.g., CCL2, CCL4, and CXCL10 belong to the family of chemokines responsible for early inflammatory chemotaxis. Also, some significant members of the TNF cytokine family were present, e.g., TNF and TNFSF11, which control and initiate a wide range of cellular activities during immediate inflammation, e.g., controlled cell death and survival, as well as systemic inflammatory response [26]. An essential cytokine family for immune cell activation and regulation is the interleukins, which are displayed within the STRING network. Both proinflammatory (IL-4, IL17A, LIF) and anti-inflammatory interleukins (IL-10RA, IL-10RB) are present [27], 28]. VEGF-A, which is not presented in the STRING network, was also present and is another example of a cytokine with essential functions in acute inflammation [29].

#### The protein expression level differences

**Figure 3:**
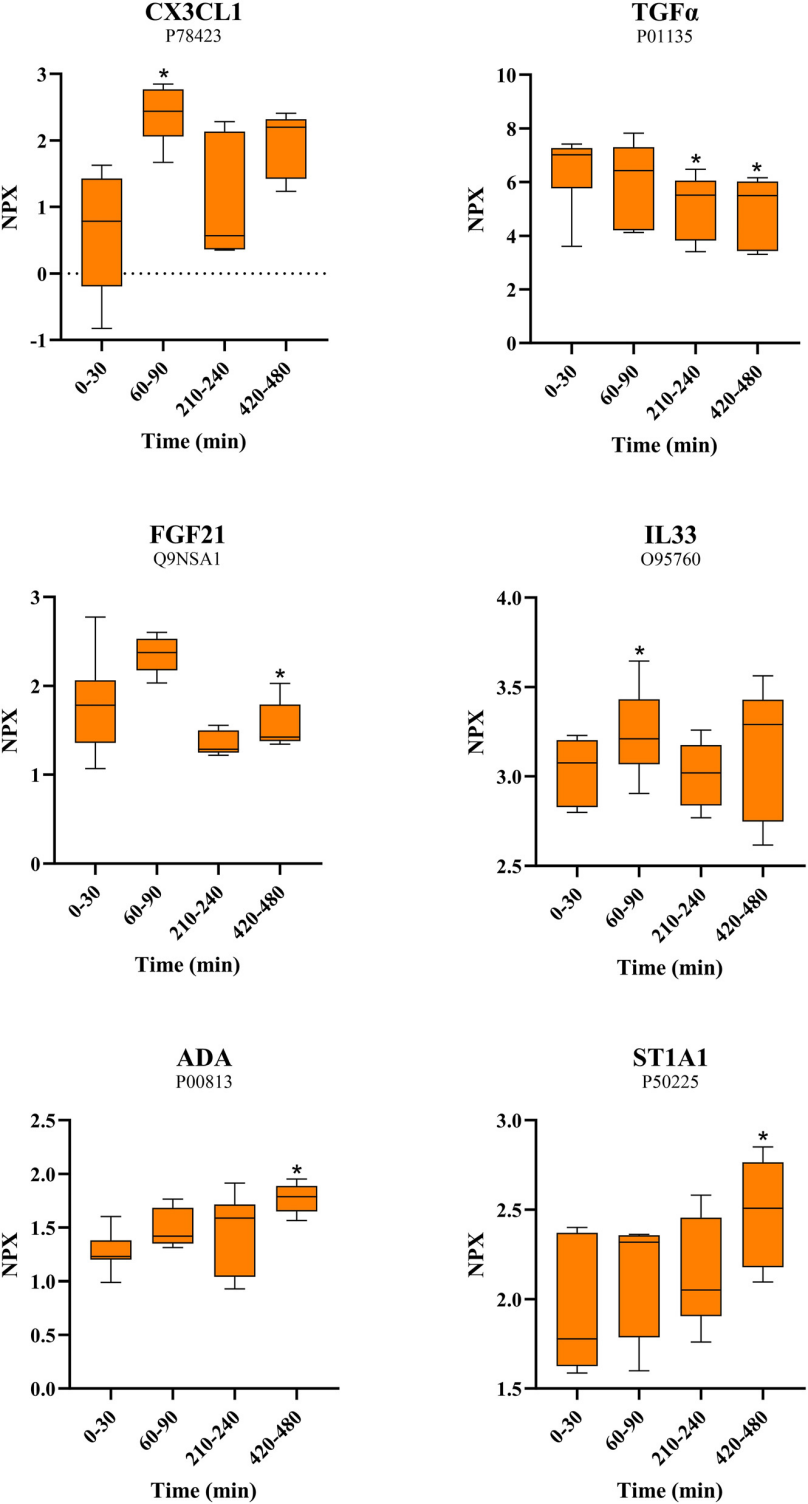
Median NPX values for the six inflammatory protein markers with significant results before adjustment. The specific protein names and UniProt IDs are shown above each figure. The vertical axes show Log2-scaled NPX values. Horizontal axes show the four time points. The boxes represent the 25th to the 75th percentiles (equal to the IQR). The whiskers show the minimum and the maximum values. *p-Value<0.05 compared to the first time point 0–30 min. Created with GraphPad Prism 10 (version 10.0.2 232) (GraphPad Software, Boston, USA).

**Figure 4:**
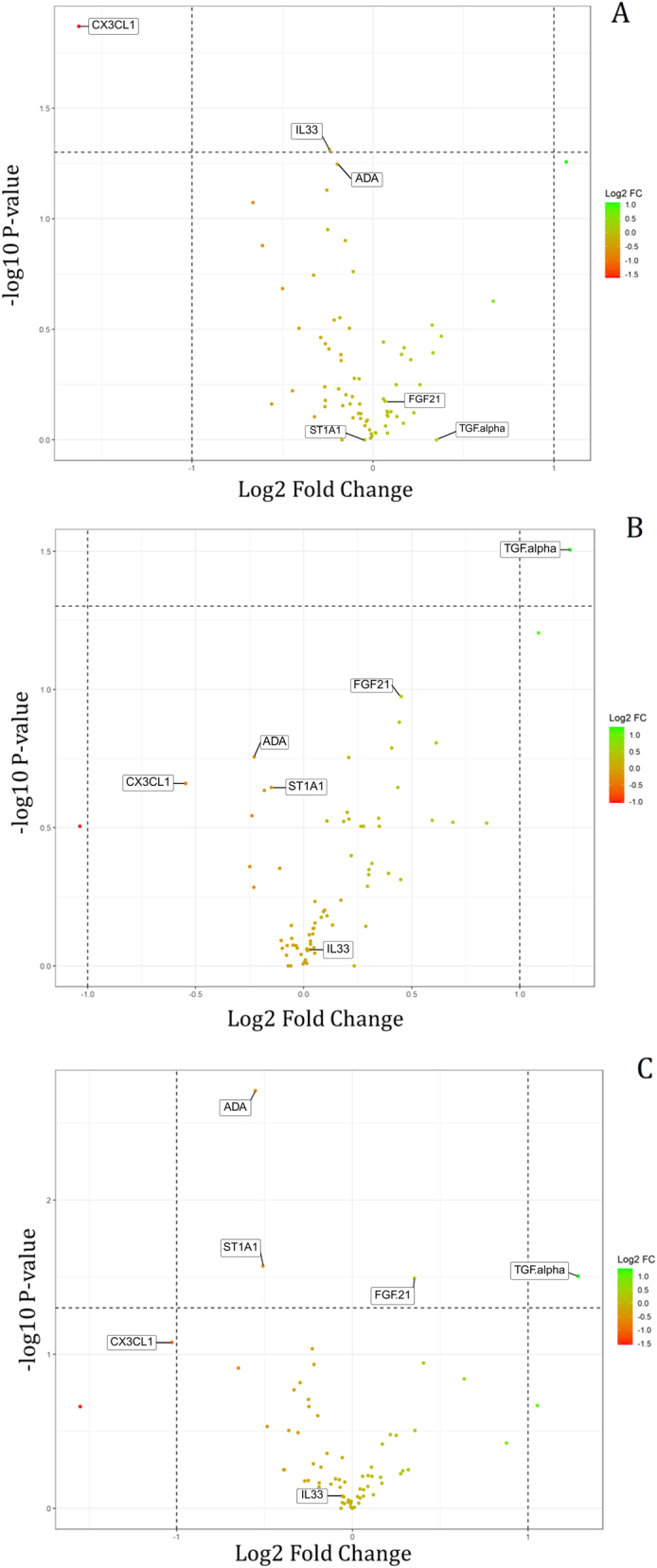
-log10 (p-Value) (nonadjusted) plotted as a function of the log2 fold change of NPX for specific proteins between time points. The higher the -log10(p-Value), the smaller the p-Value. The horizontal line indicates a p-Value<0.05. The vertical lines indicate -2- and 2-fold changes, respectively. This means that proteins displayed on the right side (more green-colored nodes) are overexpressed in the first group compared to the second group. Proteins displayed on the left side (more red-colored nodes) are overexpressed in the second group compared to the first group. A) 0–30 min vs. 60–90 min. B) 0–30 min vs. 210–240 min. C) 0–30 min vs. 420–480 min. Created with R-package *ggplot2* in R (version 4.1.3).

Six proteins showed significantly different expression levels when the group of the first time point was compared to the other three groups. When adjusted for multiple testing, the difference became insignificant. However, these preliminary data may still be of exploratory interest as this study is the first of its kind, which is why it was chosen to proceed with presenting the data. The median NPX values of the six proteins and the assessment of the relation between time points are shown in Figure 3. For the remaining proteins, analyses indicated similar expression levels during the sampling period. Figure 4 shows volcano plots illustrating the relationship between the nonadjusted p-values and fold changes for all 72 proteins for each comparison: 0–30 min vs. 60–90 min; 0–30 min vs. 210–240 min; 0–30 min vs. 420–480 min.

Of the six proteins with significant expression levels before adjustment, CX3CL1, ADA, and ST1A1 tended to increase, while TGFα and FGF21 tended to decrease. IL33 showed more fluctuating values but increased significantly from 0-30 min to 60–90 min along with CX3CL1. The proteins that showed the most significant difference between time points are CX3CL1, which increased up to three times, and TGFα, which decreased almost three times.

CX3CL1: Fractalkine. TGFα: Protransforming growth factor alpha. FGF21: Fibroblast growth factor 21. IL33: Interleukin-33. ADA: Adenosine deaminase. ST1A1=SULT1A1: Sulfotransferase 1A1.

**Figure 5:**
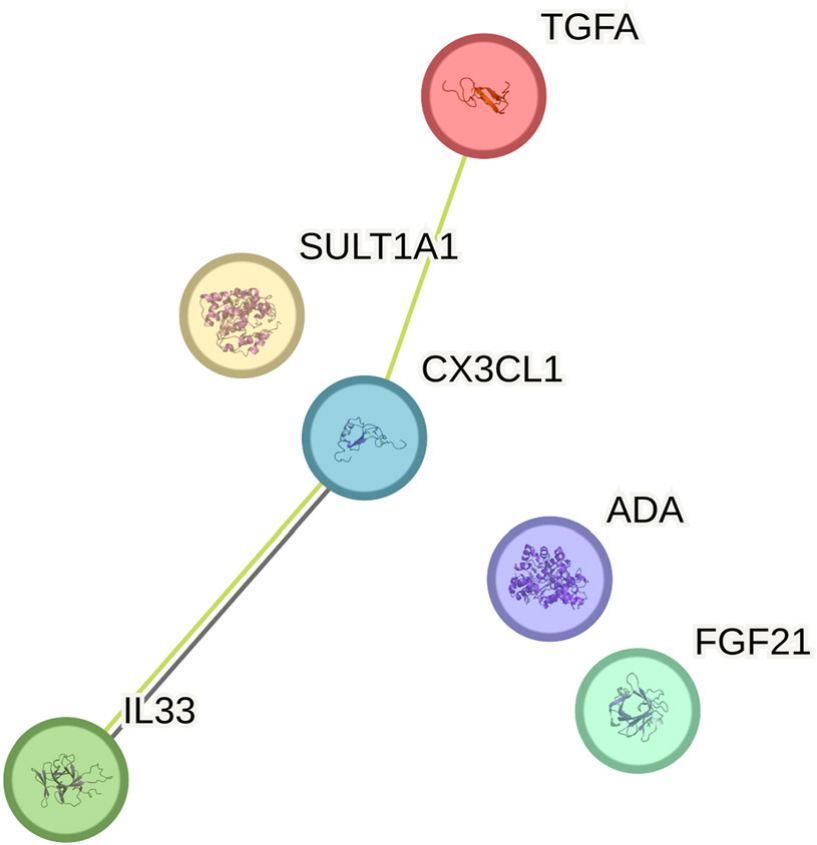
STRING network conducted including the six inflammatory protein markers with significant results before adjustment. Edges mark protein–protein associations by evidence. Black line: protein coexpression networks. Green lines: text mining protein associations (automated mining algorithms to find statistical co-occurrence of candidate gene/protein names in scientific literature). Created with STRING 12.0.

Table 1 presents the general function of the six proteins with significant changes in expression before adjustment concerning acute inflammation. Figure 5 shows a PPI STRING network with medium confidence (0.400). A PPI enrichment analysis of GO terms was conducted using STRING but with no findings.

**Table 1:** Immune-related functions for each specific protein and references to the literature.

Protein	Function related to acute inflammation	Reference
CX3CL1	Migration of T cells and natural killer cells. Facilitate the anti-inflammatory function of monocytes.	Griffith JW et al. [30]
IL33	An alarmin preproduced in the cell and released immediately upon cell injury or cell death.	Cayrol C et al. [31]
TGFα	Promotion of cell proliferation. Close relation to the group of growth factors, e.g., TGFβ.	Partridge M et al. [32] Justin Hsuan J et al. [33]
ADA	Proinflammatory response via degradation of the anti-inflammatory adenosine.	Bhagavatham SKS et al. [34]
FGF21	Metabolic regulator of mainly lipid and glucose metabolism. Some inhibition of macrophage-mediated inflammation.	Yao D et al. [35]
ST1A1	Belongs to the group of human cytosolic sulfotransferases with a vast effect on the metabolism of drugs, carcinogens, and hormones.	Berger I et al. [36]

### Ischemic metabolites

The mean concentrations of the ischemic metabolites are shown in Table 2.

**Table 2:** The mean concentrations (95 % confidence interval) of the ischemic metabolites. ^a^p-Value<0.05 compared to the first time point of 0–30 min.

Time, min	Glucose, mmol/L	Glycerol, µmol/L	Lactate, mmol/L	Pyruvate, µmol/L	Lactate/pyruvate ratio
0–30	0.4 (0.1;1.6)	58.6 (2.6;132.3)	3.1 (2.6;3.7)	156.7 (105.7;232.6)	21.0 (14.6;27.4)
60–90	0.5 (0.1;2.0)	26.4 (2.5;275.6)	3.3 (2.4;4.6)	186.6 (143.7;242.4)	18.21 (15.0;21.9)
210–240	0.4 (0.1;1.4)	35.9 (17.9;72.2)	3.3 (2.1;5.1)	169.0 (113.4;251.9)	19.5 (16.4;22.5)
450–480	0.4 (0.2;1.3)	17.8^a^ (12.9;26.6)	2.8 (1.9;4.2)	136.9 (104.7;178.9)	21.26 (16.2;26.3)

For glucose, relatively low and stable levels were detected during the sampling period. Glycerol concentrations tended decreased significantly between the first and last samples (p=0.04).

The mean L/P ratios did not exceed the ischemic cutoff level of 25. Median lactate levels reached relatively constant hyperlactatemia levels between 3 and 4 mmol/L at all time points, with pyruvate following the same pattern. Systemic lactate levels measured from arterial blood samples were in the range of 0.7–1.9 mmol/L with no specific pattern.

## Discussion

This study is the first to investigate the immediate postoperative effect of CRS with HIPEC on local tissue inflammatory and ischemic responses in a newly developed porcine model using microdialysis. As expected, an immediate inflammatory response was found in the peritoneal tissue of the abdominal wall during the first 30 min after HIPEC initiation, followed by a modest and relatively constant inflammatory response in the remaining sampling period. Relatively constant hyperlactatemia exceeding the plasma levels was seen during and after HIPEC with no significant tissue ischemia.

Only six of the 92 inflammatory protein markers investigated showed a significant difference in expression levels throughout the sampling period before adjustment for multiple testing. Of these six proteins, only TGFα, ST1A1, ST1A1, and IL33 were associated with text mining and coexpression networks. However, these interaction types do not solely mean that the proteins and the different expression levels are related [37]. No experimentally validated interactions or interactions from curated databases exist. Furthermore, the functional enrichment analysis uncovered no correlations regarding biological processes (GO-terms), and finding six proteins with different expression levels in a multiple testing set up was almost expected [38]. This may indicate that the present protein expression level differences are relatively random or coincidental.

Blood chemistry does not reveal the site of inflammation or the extent of local tissue damage, which is why the application of microdialysis for local investigation is of interest [39]. It may be difficult to compare the present results to findings from other studies due to different methodologies. A study with 26 cancer patients treated with CRS with HIPEC (mitomycin in saline 0.9 %, 90 min, 41.5 °C) studied peritoneal fluid and plasma levels of inflammatory cytokines on days 1, 2, and 3 postoperatively [5]. They found constant levels in the peritoneal fluid of the investigated pro- (e.g., TNF, FGF, VEGF, IL-6) and anti-inflammatory cytokines (e.g., IL-10), which exceeded the levels in plasma. Another study compared the cytokine response (e.g., TNF, IL-6, IL-10, IL-8, MIP-1, MCP-1) in plasma at baseline before CRS, after CRS, after HIPEC (oxaliplatin in dextrose 5 %, 30 min, 42–43 °C), and postoperatively in 20 patients, which showed significantly increased levels among all investigated cytokines in plasma during CRS followed by a minor and non-significant increase during HIPEC [40].

Of interest, the role of inflammatory markers for the tumor microenvironment in ovarian cancer has been investigated, why the local tissue responses may be just as important to evaluate as the systemic response [41]. Here, the microdialysis technique displays a beneficial tool enabling sampling from local target tissues. Several cytokines of special interest were discovered in the peritoneal tissue in the present study. Some cytokines with suggested both anti- and protumor functions, e.g., IL-33 and IL-10RA, and others with primarily antitumor effects, e.g., IL-4. Possibly, in addition to the established purpose of the treatment with CRS with or without HIPEC, inflammatory local responses may also result in treatment effects on the microscopic cancer disease.

Uncalibrated local tissue lactate levels reached 3–4 mmol/L compared to 0.7–1.9 mmol/L in plasma. *In vitro* relative recoveries for glucose, lactate, and pyruvate in 100 kilo-Dalton cutoff microdialysis catheters (as used in this study) have previously been found to exceed >90 % at a flow rate 0.3 μL/min [42]. Most commonly, hyperlactatemia is thought to be caused by tissue hypoperfusion or hypoxia. However, excessive aerobic glycolysis under stress conditions has been suggested as another and maybe even more important factor of hyperlactatemia development, which very well relates to conditions during CRS with HIPEC, and may explain the relatively nondecreased levels of pyruvate in the present study [43]. Interestingly, the mechanism for hyperlactatemia in our study does not seem to be induced by cell damage, which is indirectly illustrated by decreasing glycerol levels during and after HIPEC. Hypoxia may play a role with relatively high L/P ratios (>18), though still below the theoretical threshold for significant ischemia of <25.

The present results indicate that systemic hyperlactatemia is related and secondary to the constant local tissue hyperlactatemia. Previous studies have reported even higher plasma lactate concentrations ranging from 2.7 to 4.7 mmol/L during CRS and HIPEC [10], 12], 44]. This may be explained by a shorter and less extensive CRS procedure in this experimental setting, compared to a real clinical setting, and the investigation in young and healthy porcine tissue. The prognostic impact of plasma lactate levels and probably other factors in patients treated with CRS and HIPEC should be further evaluated in future prospective studies.

As carboplatin’s cytotoxic effects have been shown to occur after at least 12 h, the present findings are most likely based on surgical and/or hyperthermic damage [45]. It could have been valuable to perform histopathological tissue evaluation parallel to the inflammatory and ischemic metabolites responses, which should be considered in future studies. The current literature reports conflicting results on whether CRS or HIPEC primarily induces hyperlactatemia [10], 12]. Based on our results, the suggested local cytotoxic effects of hyperthermia and the chemotherapy may be seen as of minor importance, and the present results of high immediate levels of lactate and inflammatory protein markers may point to CRS being the primary cause. Also, in clinical RCTs, no difference in severe adverse events during the first 30 postoperative days has been found in CRS with HIPEC compared to CRS alone [46], 47]. CRS is a necessary part of the curative treatment of, e.g., ovarian cancer patients and the effect of the procedure is difficult to avoid. Therefore, it is highly relevant to explore the optimization potential of HIPEC, of which no standard protocol for treating, e.g., ovarian cancer patients exists [48]. Future studies incorporating histopathological tissue evaluation alongside analyzing inflammatory and ischemic metabolite responses may more effectively elaborate on these concerns.

This study was not designed to compare CRS and HIPEC, and the main limitation is the missing baseline samples before CRS. The findings may partly represent some background activity, and future studies should focus on ensuring baseline measurements and/or incorporating a control group. The inflammatory protein data were measured as NPX values, which are relative quantifications that only enable the evaluation of the inflammatory response over time. NPX value measurements depend on the respective assay, making it difficult to compare findings using different plates and techniques. For future studies on inflammatory proteins, the value of using standard concentrations (absolute concentrations, e.g., in pg/mL) vs. relative quantification (NPX values) should be considered. The applied Proximity Extension Assay is developed for studies on human samples, which may not readily be transferable to pigs. However, pigs and humans share many similarities in anatomy, physiology, and immunology, with large existing research databases showing high homology for the orthologous proteins and correlating functions [49], [50], [51]. Relative recoveries were not calculated for the ischemic metabolites, making interpreting the actual levels in the tissue difficult. Additionally, the microdialysates only reflect results from the anatomical location where they were positioned. It was believed that both surgical manipulation within the abdomen and the HIPEC procedure will result in general tissue effects across all intra-abdominal tissues; however, it should be considered in future studies to place the catheter beneath intact peritoneum close to a surgical site, e.g., the defect on the abdominal wall. Finally, the study’s exploratory nature and small sample size may limit the generalizability of the results.

## Conclusions

In conclusion, this explorative porcine study showed an immediate postoperative local tissue response of inflammatory protein markers in parallel with a relatively constant local hyperlactatemia without significant tissue ischemia during and in the short term after the HIPEC procedure. The findings suggest that the local inflammatory and ischemic responses may result mainly from a reaction to the extended CRS procedure and contribute to the debate on the value of hyperthermia in HIPEC for ovarian cancer treatment. Future studies are needed to investigate the inflammatory and metabolic tissue responses further.

## Supplementary Material

Supplementary Material
