# Supplementary material for: Immediate postoperative effects of cytoreductive surgery with hyperthermic intraperitoneal chemotherapy using carboplatin on peritoneal tissue inflammatory and ischemic responses: an explorative porcine study
Source: Pleura Peritoneum. 2025 Apr 22;10(2):69–80. doi: 10.1515/pp-2025-0001 (PMC12207598; doi:10.1515/pp-2025-0001)
Supplement: Supplementary file 1 — Supplementary Material [file j_pp-2025-0001_suppl_001.docx]

**Article title:** Immediate postoperative effects of cytoreductive surgery with hyperthermic intraperitoneal chemotherapy with carboplatin on peritoneal tissue inflammatory and ischemic responses – an explorative porcine study

**Journal name:** Clinical and Translational Oncology

**Authors:**
Elisabeth Krogsgaard Petersen*, Mats Bue, Christina Harlev, Andrea René Jørgensen, Pelle Hanberg, Lone Kjeld Petersen, Maiken Stilling.

*Corresponding author: mail: elisabethkp@clin.au.dk**; affiliation:** Department of Clinical Medicine, Aarhus University, and Aarhus Denmark Microdialysis Research (ADMIRE), Orthopedic Research Laboratory, Aarhus University Hospital, Aarhus, Denmark address: Palle Juul-Jensens Boulevard 99, J112, Aarhus, Denmark.

**Supplementary Table 1.** Median NPX (IQR) values are shown for all proteins of the Olink Target 96 Inflammation panel. The time points correspond to samples 0-30 min, 60-90 min, 210-240 min and 420-480 min.

| **Protein name UniProt ID** | **Measured frequency in the 0-30 min sample (%)** | **Median NPX (IQR)** | | | |
| --- | --- | --- | --- | --- | --- |
|  |  | **0-30 min** | **60-90 min** | **210-240 min** | **420-480 min** |
| VEGFA  P15692 | 100 | 6.2 (0.504) | 6.5 (1.17) | 6.0 (0.517) | 6.2 (0.659) |
| CD8A  P01732 | 100 | 0.57 (0.262) | 0.92 (0.301) | 0.12 (0.265) | 0.55 (0.167) |
| OPG  O00300 | 100 | 0.062 (0.168) | 0.0013 (0.433) | -0.034 (0.173) | 0.0027 (0.0340) |
| uPA  P00749 | 100 | 4.4 (0.336) | 4.8 (1.37) | 3.5 (1.73) | 2.5 (1.08) |
| MCP-1  P13500 | 100 | 11 (0.0519) | 11 (0.103) | 11 (0.125) | 11 (0.296) |
| IL-17A  Q16552 | 100 | 2.0 (0.353) | 2.2 (0.799) | 1.9 (0.453) | 1.9 (0.384) |
| CST5  P28325 | 100 | -0.52 (0.209) | -0.62 (0.744) | -0.50 (0.226) | -0.69 (0.358) |
| TGF-α  P01135 | 100 | 5.9 (1.07) | 6.4 (2.41) | 5.5 (1.58) | 5.5 (2.31) |
| MCP-4  Q99616 | 100 | 7.0 (0.857) | 7.7 (0.993) | 6.6 (0.321) | 5.6 (1.44) |
| MMP-1  P03956 | 100 | 1.9 (0.674) | 2.7 (1.55) | 1.7 (0.573) | 2.0 (0.279) |
| TNF  P01375 | 100 | 1.6 (0.316) | 1.8 (0.228) | 1.4 (0.573) | 1.7 (0.639) |
| CXCL10  P02778 | 100 | 7.0 (0.223) | 7.1 (1.63) | 7.2 (0.487) | 6.4 (0.579) |
| EN-RAGE  P80511 | 100 | 8.1 (0.752) | 7.3 (2.60) | 8.8 (2.20) | 8.6 (0.561) |
| CD40  P25942 | 100 | 1.3 (0.358) | 0.41 (1.53) | 1.2 (1.16) | 1.6 (0.814) |
| FGF-19  O95750 | 100 | 1.9 (0.200) | 2.0 (0.109) | 1.8 (0.282) | 1.8 (0.692) |
| TNF-β  P01374 | 100 | 1.5 (0.327) | 1.4 (1.12) | 1.2 (1.04) | 1.6 (0.345) |
| CXCL1  P78423 | 86 | 3.5 (0.218) | 3.4 (0.256) | 3.3 (0.600) | 3.3 (0.501) |
| CDCP1  Q9H5V8 | 86 | -0.28 (0.203) | -0.33 (0.309) | -0.39 (0.123) | -0.37 (0.220) |
| CSF-1  P09603 | 86 | 2.2 (0.443) | 2.7 (0.611) | 1.9 (0.497) | 2.8 (0.766) |
| CCL11  P51671 | 86 | 1.2 (0.0542) | 1.3 (0.0752) | 0.93 (0.472) | 1.2 (0.261) |
| FGF-21  Q9NSA1 | 86 | 1.6 (0.208) | 1.8 (0.215) | 1.3 (0.171) | 1.4 (0.141) |
| TRANCE  O14788 | 86 | 0.61 (0.197) | 0.04 (0.842) | 0.28 (2.14) | 0.80 (0.620) |
| 4E-BP1  Q13541 | 86 | 1.9 (0.212) | 1.8 (0.217) | 1.7 (0.158) | 2.1 (0.163) |
| IL4  P05112 | 86 | 0.86 (0.378) | 0.88 (0.231) | 0.96 (0.365) | 0.27 (0.868) |
| LIF  P15018 | 86 | -1.1 (0.161) | -0.87 (0.138) | -1.1 (0.378) | -0.95 (0.221) |
| DNER  Q8NFT8 | 86 | 0.24 (0.217) | 0.31 (0.377) | 0.30 (0.533) | -0.050 (0.118) |
| CCL20  P78556 | 86 | 4.2 (0.286) | 4.0 (0.559) | 4.3 (1.21) | 4.1 (0.700) |
| AXIN1  O15169 | 71 | 1.9 (0.162) | 1.9 (0.215) | 1.8 (0.343) | 1.9 (0.0498) |
| IL-10RA  Q13651 | 71 | 0.56 (0.141) | 0.55 (0.116) | 0.62 (0.144) | 0.62 (0.317) |
| CCL4  P13236 | 71 | 1.5 (0.107) | 1.6 (0.313) | 1.3 (0.427) | 1.3 (0.115) |
| IL-10RB  Q08334 | 71 | 1.2 (0.0654) | 1.2 (0.404) | 1.2 (0.337) | 1.4 (0.215) |
| NRTN  Q99748 | 71 | 0.32 (0.196) | 0.16 (0.537) | 0.045 (0.435) | 0.53 (0.221) |
| CCL25  O15444 | 71 | 0.95 (0.108) | 0.98 (0.226) | 0.87 (0.175) | 0.85 (0.0262) |
| STAMBP  O95630 | 71 | 1.0 (0.0948) | 1.1 (0.112) | 0.91 (0.510) | 0.68 (0.707) |
| CCL3  P10147 | 57 | 1.6 (0.250) | 2.1 (0.746) | 1.6 (0.292) | 1.5 (0.100) |
| TWEAK  O43508 | 57 | 0.090 (0.292) | 0.74 (0.621) | -0.42 (0.554) | 0.18 (1.19) |
| CCL28  Q9NRJ3 | 57 | 0.78 (0.171) | 0.87 (0.312) | 0.86 (0.431) | 0.94 (0.514) |
| IL-12B  P29460 | 57 | 1.1 (0.614) | 0.94 (1.04) | 1.2 (0.543) | 0.51 (0.418) |
| CASP-8  Q14790 | 57 | 2.0 (0.129) | 2.2 (0.591) | 1.9 (0.206) | 1.8 (0.378) |
| CXCL5  P42830 | 57 | 1.2 (0.437) | 1.1 (0.568) | 1.1 (0.479) | 1.6 (1.07) |
| OSM  P13725 | 57 | 0.51 (0.577) | 0.93 (0.981) | 0.41 (0.240) | 1.3 (0.242) |
| TRAIL  P50591 | 57 | 0.052 (0.140) | 0.0075 (0.155) | -0.037 (0.368) | 0.020 (0.0923) |
| CCL19  Q99731 | 57 | 1.4 (0.295) | 1.4 (0.552) | 0.94 (0.523) | 1.3 (0.327) |
| IL6  P05231 | 43 | 2.7 (0.0868) | 2.7 (0.131) | 2.5 (0.205) | 2.7 (0.156) |
| IL-20  Q9NYY1 | 43 | 0.46 (0.0596) | 0.40 (0.148) | 0.36 (0.403) | 0.38 (0.277) |
| CD6  P30203 | 43 | 0.53 (0.199) | 0.11 (1.07) | 0.48 (0.228) | 0.42 (0.513) |
| ARTN  Q5T4W7 | 43 | 1.3 (0.500) | 1.5 (0.508) | 1.1 (0.544) | 1.6 (0.520) |
| IFN-γ  P01579 | 43 | 1.9 (0.771) | 2.6 (0.569) | 1.4 (0.842) | 1.9 (0.677) |
| MCP-3  P80098 | 29 | 2.2 (0.281) | 2.5 (0.113) | 2.2 (0.192) | 2.5 (0.191) |
| GDNF  P39905 | 29 | 2.8 (0.452) | 3.2 (0.408) | 2.6 (0.260) | 3.1 (0.229) |
| CD5  P10147 | 29 | 0.59 (0.152) | 0.67 (0.203) | 0.49 (0.352) | 0.70 (0.0254) |
| IL-15RA  Q13261 | 29 | 2.9 (0.126) | 2.9 (0.172) | 2.8 (0.155) | 3.0 (0.205) |
| LAP TGF-β-1  P01137 | 29 | 1.5 (0.074) | 1.5 (0.129) | 1.6 (0.207) | 1.6 (0.171) |
| ST1A1  P50225 | 29 | 2.1 (0.285) | 2.3 (0.386) | 2.1 (0.366) | 2.5 (0.417) |
| TNFSF14  O43557 | 29 | 1.6 (0.0909) | 1.6 (0.0984) | 1.4 (0.106) | 1.7 (0.165) |
| FGF-23  Q9GZV9 | 29 | 2.2 (0.149) | 2.4 (0.253) | 2.2 (0.0471) | 2.1 (0.146) |
| CD244  Q9BZW8 | 29 | 3.3 (0.257) | 3.5 (0.230) | 3.3 (0.367) | 3.2 (0.537) |
| IL7  P13232 | 29 | 0.25 (0.075) | 0.16 (0.308) | 0.26 (0.313) | -0.037 (0.131) |
| IL33  O95760 | 29 | 3.1 (0.132) | 3.2 (0.184) | 3.0 (0.239) | 3.3 (0.421) |
| SCF  P21583 | 29 | 2.0 (0.249) | 2.1 (0.431) | 1.9 (0.246) | 2.0 (0.470) |
| IL18  Q14116 | 29 | 1.5 (0.280) | 1.7 (0.208) | 1.2 (1.15) | 1.8 (0.145) |
| IL-18R1  Q13478 | 14 | 2.4 (0.282) | 2.6 (0.550) | 2.2 (0.141) | 2.7 (0.115) |
| HGF  P14210 | 14 | 0.89 (0.271) | 0.82 (0.504) | 0.94 (0.254) | 1.0 (0.305) |
| MCP-2  P80075 | 14 | 2.6 (0.245) | 2.7 (0.591) | 2.6 (0.519) | 2.6 (0.439) |
| SIRT2  Q8IXJ6 | 14 | 3.0 (0.181) | 2.9 (0.339) | 2.7 (0.585) | 3.0 (0.736) |
| CXCL6  P80162 | 14 | 0.59 (0.173) | 0.67 (0.575) | 0.51 (0.498) | 0.72 (0.318) |
| IL5  P05113 | 14 | 2.1 (0.369) | 2.2 (0.285) | 2.0 (0.609) | 2.4 (0.180) |
| ADA  P00813 | 14 | 1.5 (0.270) | 1.4 (0.244) | 1.6 (0.456) | 1.8 (0.0846) |
| MMP-10  P09238 | 14 | 1.2 (0.105) | 1.2 (0.268) | 1.1 (0.344) | 1.4 (0.171) |
| IL-24  Q13007 | 14 | 2.1 (0.329) | 1.9 (0.889) | 2.0 (0.456) | 2.8 (1.43) |
| IL10  P22301 | 14 | 1.6 (0.301) | 1.6 (0.835) | 1.6 (0.566) | 1.4 (0.684) |
| CX3CL1  P78423 | 0 | 1.4 (0.806) | 2.4 (0.425) | 0.57 (1.30) | 2.2 (0.622) |
| **Excluded proteins from statistical analysis** | | | | | |
| FIt3L  P49771 |  | --- | --- | --- | --- |
| IL8  P10145 |  | --- | --- | --- | --- |
| IL.17C  Q9P0M4 |  | --- | --- | --- | --- |
| CXCL11  O14625 |  | --- | --- | --- | --- |
| IL.20RA  Q9UHF4 |  | --- | --- | --- | --- |
| CXCL9  Q07325 |  | --- | --- | --- | --- |
| IL.2RB  P14784 |  | --- | --- | --- | --- |
| IL.1.α  P01583 |  | --- | --- | --- | --- |
| IL2  P60568 |  | --- | --- | --- | --- |
| TSLP  Q969D9 |  | --- | --- | --- | --- |
| SLAMF1  Q13291 |  | --- | --- | --- | --- |
| FGF.5  P12034 |  | --- | --- | --- | --- |
| LIF.R  P42702 |  | --- | --- | --- | --- |
| IL.22.RA1  Q8N6P7 |  | --- | --- | --- | --- |
| PD.L1  Q9NZQ7 |  | --- | --- | --- | --- |
| β.NGF  P01138 |  | --- | --- | --- | --- |
| IL13  P35225 |  | --- | --- | --- | --- |
| CCL23  P55773 |  | --- | --- | --- | --- |
| TNFRSF9  Q07011 |  | --- | --- | --- | --- |
| NTF3  P20783 |  | --- | --- | --- | --- |
